# Occurrence of major mycotoxins in noncorn forages for dairy cattle: A survey of silages and hays from Italian farms

**DOI:** 10.3168/jdsc.2025-0913

**Published:** 2025-12-04

**Authors:** Gabriele Rocchetti, Alessandro Catellani, Maddalena Canossa, Michela Errico, Federico Froldi, Marco Lapris, Carmelo Mastroeni, Valentina Novara, Antonio Gallo

**Affiliations:** Department of Animal Science, Food, and Nutrition (DiANA), Università Cattolica del Sacro Cuore, 29122 Piacenza, Italy

## Abstract

The occurrence of mycotoxins in ruminant feeds has been traditionally linked to corn silage, but alternative forages such as cereal straw, grass silages, sorghum, and legume hays may also contribute to exposure. We investigated the main regulated mycotoxins in 977 forage samples from Italian farms, including 642 silages (wheat, grasses, sorghum) and 335 hays (wheat straw, grasses, alfalfa). Samples were analyzed for deoxynivalenol, zearalenone (ZEN), and total fumonisins (FUM) using accredited methods. Noncorn forages were not exempt from contamination, showing variable prevalence and concentrations depending on forage type and botanical origin. Deoxynivalenol was the most prevalent, ZEN showed moderate prevalence with rising concentrations in recent years, and FUM occurred less frequently but with occasional and not significant high levels. These findings highlight the need for continuous monitoring of all forage types, suggesting that mycotoxin risk assessment in dairy diets should extend beyond corn silage.

## Highlights

• Noncorn forages are sensitive to mycotoxin contamination.
• The most prevalent mycotoxin across all annual periods was DON.
• Total fumonisin occurrence in noncorn forages was generally lower than in corn-based feeds.
• A moderate rise in ZEN concentrations was observed in sorghum silages and straw.
• Surveillance of the full forage spectrum could increase feed safety.

## Graphical Abstract

**Figure:**
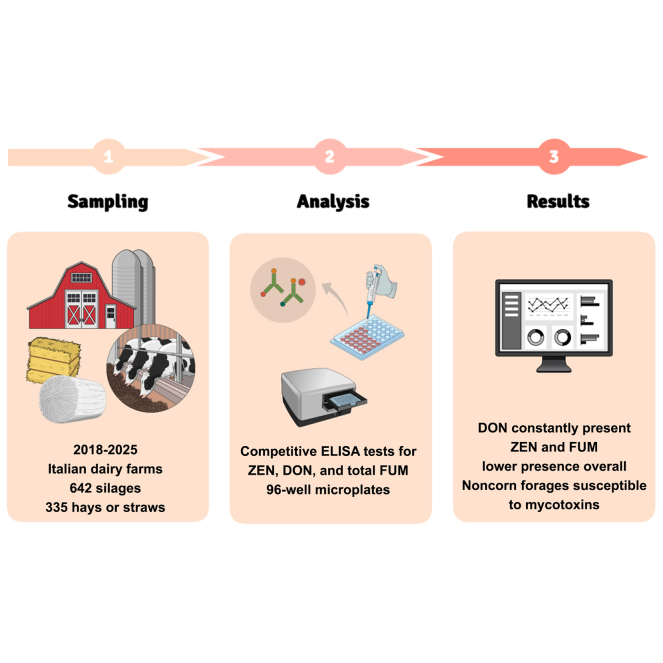
**Summary:** In this study, 977 noncorn forage samples from dairy farms located mainly in Northern Italy were analyzed over a 7-year period; samples included 642 silages and 335 hays or straws. Deoxynivalenol (DON), zearalenone (ZEN), and total fumonisins (FUM) were quantified using competitive enzyme-linked immunosorbent assay (ELISA) methods. Deoxynivalenol was the most frequently detected mycotoxin (48% of samples), with particularly high concentrations in straw and mixed-cereal silages. Zearalenone showed lower overall prevalence but reached higher levels in sorghum silages and straw. Total fumonisins were less common across all forage types, with the highest occurrence in sorghum silages and negligible contamination in legume hays. Climatic variability across years, especially the wetter conditions recorded in 2018 through 2019, contributed to the observed differences in mycotoxin occurrence. Overall, the findings confirm that noncorn forages are susceptible to Fusarium mycotoxins, with contamination patterns influenced by botanical composition, environmental conditions during growth and harvest, and farm-level conservation practices. Image generated using the design program Mind the Graph (https://mindthegraph.com/; accessed Oct. 8, 2025).

Mycotoxin contamination represents a well-documented issue in animal production, with important implications for both livestock health and food safety (Akinmoladun et al., 2025). Mycotoxins are toxic secondary metabolites produced mainly by fungi belonging to the genera *Aspergillus*, *Fusarium*, and *Penicillium* (Gallo et al., 2015). Their occurrence in forages used for ruminant feeding can lead to a wide range of consequences, from subclinical effects such as reduced feed intake, impaired feed efficiency, and immune or reproductive disturbances, to more severe outcomes under chronic exposure (Yang et al., 2020; Xu et al., 2022). Scientific literature and official monitoring programs have strongly focused on corn and whole-plant corn silage, which are recognized as highly susceptible substrates for fungal colonization (Ogunade et al., 2018; Ghilardelli et al., 2022). However, dairy cattle diets also rely extensively on alternative forages, including small-grain cereal silages, sorghum, grasses, and hay or straw from cereals and legumes such as alfalfa (Burke et al., 2007; Cattani et al., 2017; Xia et al., 2025). Despite their widespread use across Italian and European farms, knowledge on mycotoxins contamination in these noncorn forages remains limited and fragmented. Previous reports have highlighted the presence of *Fusarium*-derived toxins, such as deoxynivalenol (**DON**), zearalenone (**ZEN**), fumonisins (**FUM**), and T-2/HT-2 toxins, in cereal straws and minor grains, as well as aflatoxins and ochratoxin A in hays subjected to inadequate storage conditions (Yu and Pedroso, 2023; Gozzi et al., 2024). Contamination levels can be influenced by several factors, including forage species, harvesting stage, climatic conditions during drying or ensiling, and conservation practices at the farm level (Manni et al., 2022).

The present study aimed to systematically investigate the occurrence of major regulated mycotoxins in noncorn forages, analyzing a large dataset of 977 samples collected from dairy farms located in Northern Italy, mainly in the forage-producing lowland areas of Piedmont, Lombardy, Veneto, and Emilia-Romagna. The temporal distribution was not fixed across years; sample submissions occurred throughout the year, with variable proportions in each quadrimester depending on forage availability and routine monitoring practices. Across 2018 through 2025, 28%–66% of samples were delivered in January through April, 18%–37% in May through August, and 28%–51% in September through December. Climatic conditions of the mentioned forage-producing areas were summarized for the investigation (ISTAT, 2024; SNPA, 2025). Annual mean temperatures ranged between 14.2°C and 15.3°C, with minimum and maximum means spanning 9.4°C to 10.5°C and 18.6°C to 19.9°C, respectively. Relative humidity varied from 71.7% to 78.5%, and cumulative precipitation from 528 to 1,104 mm. Specifically, 2018 and 2019 were warm and humid years with high rainfall (815–844 mm), 2020 and 2022 were markedly drier (528–653 mm), 2023 showed moderate precipitation (717 mm), and 2024 recorded the highest rainfall of the series (1,104 mm).

The dataset includes 642 silages (mainly wheat, grasses, and sorghum) and 335 hays (primarily wheat straw, grasses, and legumes such as alfalfa). The silages mainly consisted of wheat (n = 202), mixed grasses and autumn cereals (n = 160), sorghum (n = 121), triticale (n = 42), alfalfa and soybean mixtures (n = 38), ryegrass (n = 38), barley (n = 33), rye (n = 7), and oat (n = 1). The hays and straws included wheat straw (n = 124), grass mixtures (n = 102), grass-legume mixtures (n = 33), alfalfa hay (n = 30), wheat hay (n = 20), ryegrass hay (n = 16), oat hay (n = 7), triticale hay (n = 1), barley hay (n = 1), and millet hay (n = 1). The category “mixed-cereal silages” included autumn-winter forage mixtures mainly composed of small-grain cereals (e.g., wheat, triticale, barley, and ryegrass). Forage sampling followed the Laboratorio di Analisi Zootecniche S.a.s. (Gonzaga, Italy; https://www.lazoovet.it/) internal procedure A1-IO-ACC rev.2 and the principles of EU Regulation 152/2009 (European Commission, 2009). Silage samples were collected using a multipoint random approach from at least 5 positions along the silage face or from multiple cores in wrapped bales; hay samples were taken with a mechanical corer from different bales and depths to avoid leaf-stem segregation. Subsamples were combined into a single composite sample per lot, with a minimum representative quantity of approximately 1.5 kg for silages and 0.5 to 1 kg for hays. For mycotoxin determination, larger composite masses were occasionally prepared to improve representativeness before milling. Samples were sealed in robust polyethylene bags (nonbiodegradable), kept refrigerated (4°C–10°C) or frozen when necessary, transported in insulated boxes with cooling packs if delivery exceeded 1 d, and homogenized upon arrival at the laboratory for analysis. For statistical purposes, the results of mycotoxin analyses were considered collectively, combining silages and hays into a single dataset of 977 samples. Mycotoxin concentrations were expressed on a DM basis.

From 2018 to 2025, ZEN, DON, and total FUM were determined by competitive ELISA (RIDASCREEN and Tecna/Eurofins) at the Laboratorio di Analisi Zootecniche. All forage samples were numbered, labeled, and milled to 0.5 mm. Silages were first homogenized with a blade cutter, then dried at 65°C for approximately 16 h before milling; hay moisture was measured with a thermobalance. For ZEN and FUM, 5.00 ± 0.05 g (± SD) of sample were extracted with methanol-water (70:30, vol/vol) plus NaCl; for DON, 5.00 g were extracted with 50 mL of distilled water. Extracts were clarified by sedimentation, filtration through Whatman No. 1 paper (Merck Life Science S.r.l., Milan, Italy), and pH adjustment (6.5–7.5). Solid-phase extraction (**SPE**) purification was applied to ZEN (Romer MycoSep AlfaZon) and DON (Romer MycoSep Trich), while FUM was analyzed without SPE. Purified extracts were stored at +4°C for short-term analysis or −15°C for up to 5 d. Enzyme-linked immunosorbent assay plates precoated with specific antibodies were processed according to kit protocols, including incubation with horseradish peroxidase conjugates, washing, 3,3′,5,5′-tetramethylbenzidine development, and acid stopping. Optical density at 450 nm was used to construct %B/B_0_ standard curves (where B represents the absorbance of the standard or sample and B_0_ that of the zero standard) and interpolate sample concentrations via cubic spline regression (RidaSoft, R-Biopharm Italia S.r.l., Milan, Italy), adjusting for sample weight and dilution. Each analytical batch included duplicate quality control materials and was monitored with control charts; the method was externally validated through participation in the 2023 BIPEA 31E (https://www.bipea.org/feed-2/) mycotoxin proficiency test on forage matrixes.

All statistical analyses were performed using R (version 4.4.1; R Foundation for Statistical Computing, Vienna, Austria) within the RStudio environment. To assess potential temporal trends in mycotoxin contamination, we applied weighted linear regression (**WLR**) models, where annual mean concentrations of each mycotoxin (DON, ZEN, and total FUM) were regressed against sampling year. The number of samples analyzed per year showing quantitative values greater than the limit of quantification (**LOQ**) was used as a weighting factor in order to account for the unequal sampling effort across years. Model fit and statistical significance (*P* < 0.05) of regression coefficients were evaluated using standard diagnostics (*t*-tests for coefficients, *F*-test for overall model fit, and R^2^ values for variance explained). Graphical visualization of the data was performed using the ggplot2 package, including scatterplots of annual mean concentrations scaled by sample size and regression lines with 95% CI.

A total of 977 forage samples (silages and hays) were analyzed over a 7-yr period for the presence of ZEN, DON, and total FUM. Table 1 summarizes the number of samples analyzed per forage type, the proportion of positive samples above the LOQ, and the mean concentration of positive samples expressed in µg/kg DM. Overall, DON was the most frequently detected mycotoxin, and FUM showed lower prevalence across all forage types. Variations in occurrence and concentration were observed depending on the botanical composition and type of forage. As a general consideration, DON was the most prevalent mycotoxin, detected in the 48% of the analyzed samples, with the highest concentrations observed in straw (average: 950 µg/kg DM) and mixed-cereal silages (average: 1,358 µg/kg DM). Zearalenone showed a lower prevalence overall, but sorghum silages and straw had notably higher positive rates and concentrations (Table 1). Total FUM were less frequently detected, with moderate average concentration values; the highest occurrence was observed in sorghum silages, while legume hay showed minimal contamination. These results highlight that noncorn forages are sensitive to mycotoxin contamination, and both forage type and botanical composition can potentially affect the risk of exposure. The differential occurrence of DON, ZEN, and FUM across forage types can be partly explained by the ecological niche of their main producing fungi. *Fusarium graminearum* and related species, responsible for DON and ZEN biosynthesis, are strongly associated with cereal residues and straw, which explains the high positivity in wheat straw and mixed-cereal silages (Gozzi et al., 2024). In contrast, FUM are typically produced by *F. verticillioides* and *F. proliferatum*, fungi better adapted to warm and dry conditions, which may account for their sporadic but sometimes severe contamination in sorghum silages (del Palacio et al., 2016). Legume-based forages, by comparison, tend to exhibit lower contamination rates, likely due to differences in plant physiology and structural composition, which reduce susceptibility to *Fusarium* colonization (Munkvold et al., 2021). These species-specific interactions highlight how both botanical origin and ensiling dynamics shape the mycotoxin risk profile in noncorn forages.

**Table 1:** Occurrence (% of positive samples) and contamination levels in noncorn forages (2018–2025)^1^

Mycotoxin, by forage type	Number of samples	% Positive (>LOQ)^2^	Average (μg/kg DM)	Median (μg/kg DM)	Maximum (μg/kg DM)
ZEN					
All	540	29	128 ± 151	68	943
Mixed-cereal silages^3^	270	21	83 ± 83	45	380
Sorghum silage	76	66	181 ± 177	124	840
Legume silage	18	56	61 ± 28	60	122
Grass hay	72	24	135 ± 169	64	598
Straw	55	27	180 ± 235	107	943
Legume hay	49	20	99 ± 107	44	335
DON					
All	903	48	929 ± 3,037	281	46,000
Mixed-cereal silages	452	39	1,358 ± 4,420	292	46,000
Sorghum silage	108	44	499 ± 526	283	2,676
Legume silage	37	43	221 ± 113	191	529
Grass hay	135	53	403 ± 539	227	3,147
Straw	119	84	950 ± 2,130	319	16,486
Legume hay	52	44	551 ± 638	456	3,182
Total FUM					
All	491	20	1,005 ± 697	765	4,501
Mixed-cereal silages	253	17	1,092 ± 867	824	4,501
Sorghum silage	70	33	1,024 ± 619	790	3,182
Legume silage	26	23	1,115 ± 792	783	2,673
Grass hay	47	13	796 ± 314	655	1,395
Straw	73	25	801 ± 302	687	1,727
Legume hay	22	5	1,098	NA^4^	1,098

1 Results are expressed as average values in positive samples ± SD. The median and maximum concentration values are also reported.

2 LOQ values were as follows: ZEN >25 μg/kg; DON >100 μg/kg; total FUM >500 μg/kg.

3 Silages produced from autumn–winter forage mixtures, mainly composed of small-grain cereals (wheat, triticale, barley, and ryegrass).

4 NA = not available.

The annual analysis of 540 forage samples for ZEN (2018–2025) showed moderate variability in both prevalence and concentration over the years. Positivity rates fluctuated between 18% and 44%, with the highest proportion of positive samples observed from 2019 to 2020 (44%). Mean concentrations of positive samples ranged from 55 µg/kg in 2022 to 2023 to 193 µg/kg in 2024 to 2025, indicating a marked increase in the last year of observation (Figure 1). Notably, despite some fluctuations in prevalence, the upward trend in mean concentration during 2024 to 2025 suggested that ZEN contamination became more pronounced, particularly in certain forage types, potentially reflecting climatic or agronomic variations. Deoxynivalenol was consistently the most prevalent mycotoxin across all annual periods, with positivity rates ranging from 42% to 62%. The 2018 to 2019 season showed an unusually high mean concentration of 4,099 µg/kg, markedly higher than all subsequent years. After this peak, prevalence remained relatively stable (42%–46%), while mean concentrations of positives decreased substantially to 423 to 698 µg/kg, with a gradual increase in the last 2 yr (2023–2025). This pattern indicates that although DON is widely distributed in noncorn forages, extreme contamination events may occur sporadically, likely influenced by environmental conditions or crop management. Total FUM were generally less prevalent, with annual positivity rates ranging from 9% to 40%. Low sample numbers in the early years (2018–2020) limit trend interpretation. However, from 2020 onward, there was a notable increase in both prevalence and concentration, peaking in 2023 to 2024, with 40% positive samples at a mean concentration of 838 µg/kg. Interestingly, the 2024 to 2025 period showed a decline in prevalence (11%) but an increase in mean concentration of positives to 1,439 µg/kg, suggesting that fewer samples were contaminated, but those affected exhibited higher toxin loads. This sporadic distribution is consistent with FUM occurrence in noncorn forages, which is generally lower than in corn-based feeds.

**Figure 1:**
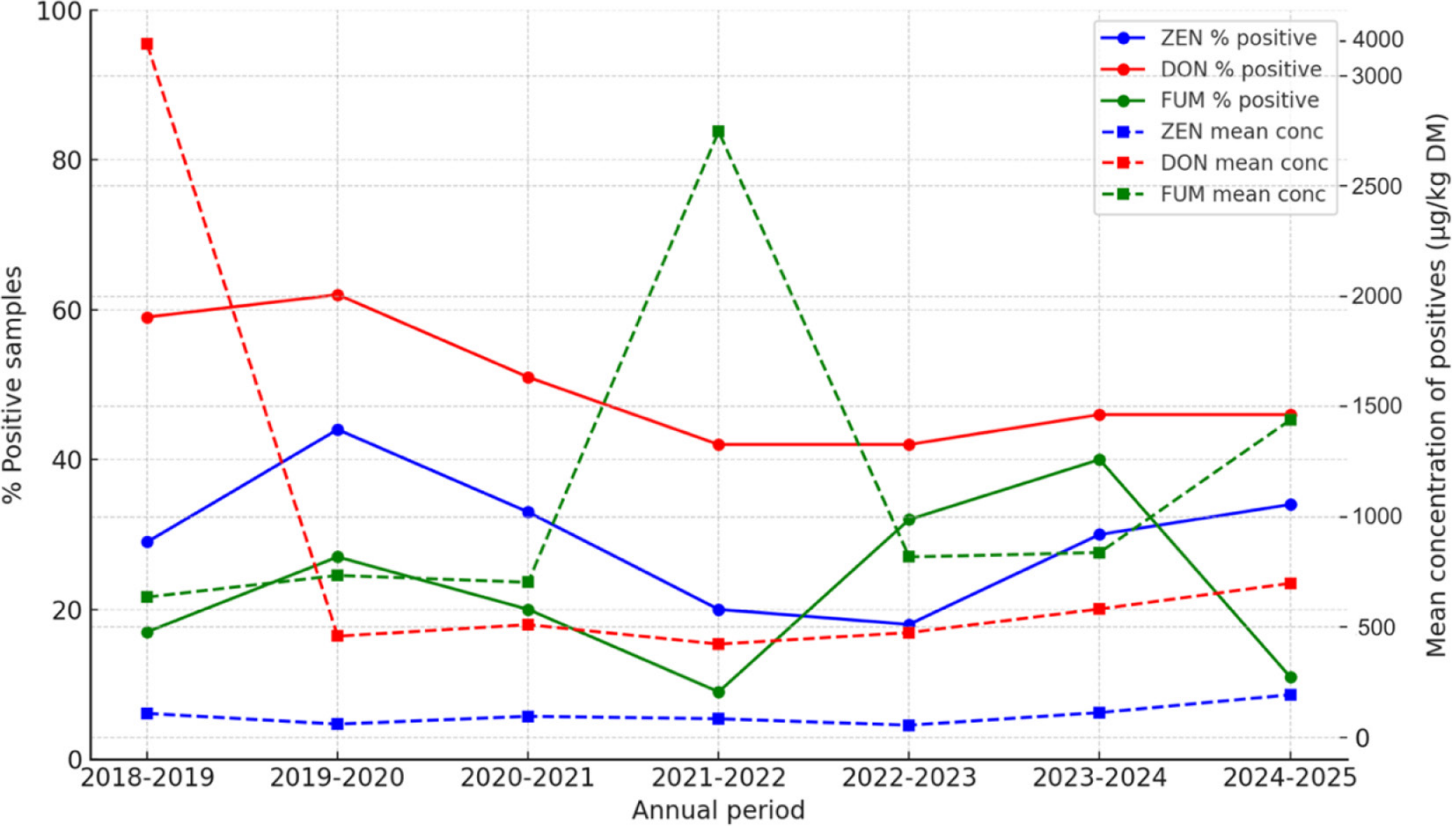
Annual trends in the prevalence (% positive) and mean concentration (conc; µg/kg DM) of zearalenone (ZEN), deoxynivalenol (DON), and total fumonisins (FUM) in noncorn forages analyzed from 2018 to 2025. Solid lines indicate percent of positive samples above the LOQ, and dashed lines indicate mean concentration of positive samples.

To investigate potential temporal trends in silage contamination, we applied WLR models, where annual mean concentrations of each mycotoxin (from 2018–2019 up to 2024–2025) were regressed against sampling year. Weights were assigned based on the number of samples analyzed per year, in order to account for the unequal sample size across years and provide a more robust estimate of overall tendencies. The weighted regression curves for each mycotoxin are reported in Figure 2. The regression model indicated a negative slope for DON concentrations over the study period (β = −262 µg/kg per year), suggesting a possible decline. However, the association was not significant (*P* = 0.236), and the explained variance remained scarce (R^2^ = 0.27). These results imply that DON contamination did not follow a consistent linear trend across the investigated years. For ZEN, the model estimated a positive slope corresponding to an annual increase of approximately +19 µg/kg (β = 19.5 µg/kg per year). The association approached significance (*P* = 0.073), with the model explaining nearly half of the weighted variance (R^2^ = 0.51). Although the overall regression was not significant, the highest ZEN concentrations were observed in the last sampling years (i.e. 2024–2025), particularly in sorghum silages and straw, indicating that these forage types contributed most to the apparent upward pattern. In contrast, total FUM concentrations did not show any evidence of temporal variation. The regression slope was slightly positive (+58 µg/kg per year), but highly uncertain, and not significant (*P* = 0.701). The explained variance was negligible, indicating that year-to-year changes in FUM levels were not captured by a linear trend model. Therefore, the weighted regression approach confirmed the complexity of interpreting temporal dynamics in mycotoxin contamination. While DON showed a numerical tendency to decline, the lack of significance reflects the nature of *Fusarium* epidemics, which are strongly dependent on seasonal climatic factors such as rainfall during flowering and pre-harvest periods (Chandelier et al., 2011). Conversely, the near-significant upward trajectory of ZEN is noteworthy because it suggests a progressive increase in contamination pressure that may mirror shifts in rainfall distribution and relative humidity during forage harvest, as also observed by Moraes et al. (2023). The absence of any consistent pattern for FUM further underlines that these toxins are likely driven by episodic, localized events rather than long-term regional trends. Such variability emphasizes the importance of adopting flexible monitoring strategies rather than assuming predictable trajectories. A limitation of this survey is the heterogeneous sampling across years and forage types, which may have affected trend estimates despite weighted regression. The focus on DON, ZEN, and total FUM excluded other relevant toxins, such as T-2/HT-2 or emerging *Fusarium* metabolites. Additionally, ELISA assays were used for rapid, cost-effective quantification, offering high throughput and applicability to complex feeds, but they are subject to cross-reactivity, matrix effects, and higher detection limits compared with the more accurate liquid chromatography-tandem MS analysis. Thus, results should be seen as semiquantitative, yet sufficient for monitoring trends and relative differences. Despite these limitations, the dataset of approximately 1,000 samples over 7 yr provides robust evidence that mycotoxin risk extends beyond corn silage. To summarize, this comprehensive survey revealed that ZEN was detected on average in approximately 25% of all forage samples. The mean concentration of ZEN was approximately twice as high in grasses compared to legumes, both in silages and hays (the 56% positivity observed in legume silages was not considered because of the very small sample size, n = 18). Within the grass group, straw exhibited the highest mycotoxin load. Over the years, ZEN was found in 30% to 40% of samples, with concentrations ranging from 60 to 110 µg/kg. The period from July 2024 to June 2025 showed the largest increase, with mean concentrations nearly doubling to 193 µg/kg. Deoxynivalenol was present in approximately 50% of samples, with no major differences between forage types, except for wheat straw, which stood out both for the proportion of positive samples and the mean toxin concentration. Silages of mixed grasses also contained relatively high DON levels, although they were less widely distributed compared to straw. The 2018 to 2019 season was notable for extremely high DON concentrations, while in subsequent years prevalence remained stable and mean concentrations gradually increased. The unusually high DON concentrations observed in 2018 to 2019 coincided with years characterized by elevated humidity and precipitation across the main forage-producing regions (relative humidity 73%–75%; precipitation 815–844 mm), whereas the years 2020 to 2022 were markedly drier (528–653 mm). Although annual climatic means cannot fully capture crop-specific exposure during critical phenological phases, the wetter conditions of 2018 through 2019 were consistent with environmental scenarios known to promote *F. graminearum* development and DON accumulation. Total FUM were detected in approximately 20% of all forages, with legume hays appearing largely free of contamination. The number of samples analyzed prior to early 2023 was too limited to assess temporal trends; however, from 2023 onward, FUM were more frequently detected, albeit at relatively low concentrations.

**Figure 2:**
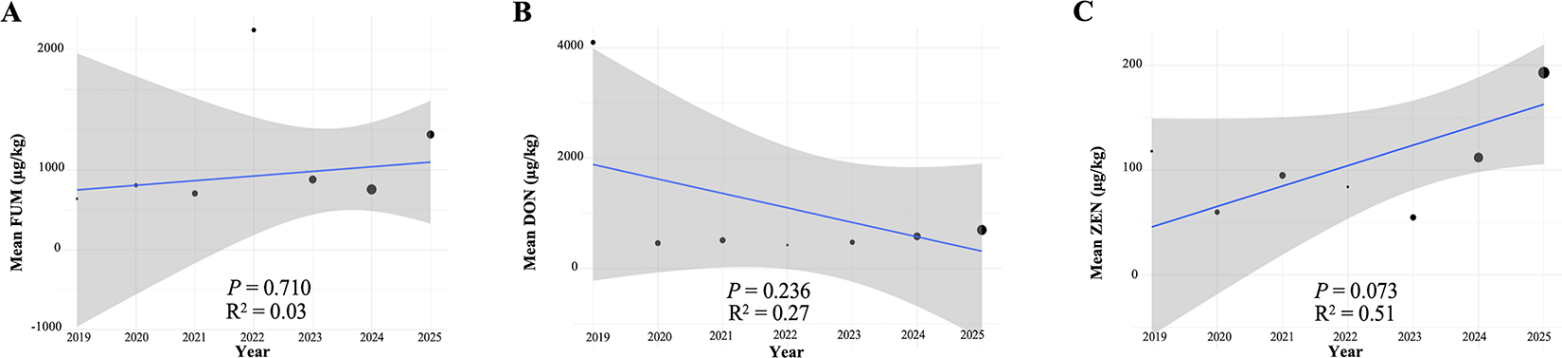
Weighted regression curves for each mycotoxin group: (A) total FUM, (B) DON, and (C) ZEN resulting from the WLR statistical approach. Black dots indicate annual mean mycotoxin concentrations, with dot size scaled according to the number of samples above the LOQ per year. The blue line shows the weighted linear regression fit, and the shaded area represents the 95% CI of the regression model.

From a practical standpoint, these findings have implications for dairy cattle feeding strategies and feed safety assessment. It is well known that *Fusarium*-produced mycotoxin contamination typically has a negative effect on cow's performance, as reviewed by Gallo et al. (2022), even when the levels at which these effects can be detected in commercial farms are still questionable and largely discussed by the scientific community and dairy technicians. Several authors (Seeling et al., 2006; Jovaišienė et al., 2016; Hartinger et al., 2022) reported that contamination levels under the recommendation proposed by the European Food Safety Authority (EFSA) could have negative effects on milk production and quality, reproductive performances, and animal health status. Among these, in vivo trials investigating the effect of DON (Gallo et al., 2020; Catellani et al., 2023), ZEN (Seeling et al., 2006; Jovaišienė et al., 2016; Catellani et al., 2025), and FUM (Gallo et al., 2020; Hartinger et al., 2022) recently reported a negative effect on cows' performance at the threshold of 0.5 to 1.0 mg/kg, 0.3 to 0.35 mg/kg, and 1.0 to 2.0 mg/kg, respectively. Based on the field aforementioned contamination levels and diet inclusions reported for specific forages in Gallo et al. (2022), noncorn forages could contribute to 5.95% ± 6.5%, 15.34% ± 8.76%, and 10.52% ± 6.34% of the proposed risk threshold for ZEN, DON, and FUM, respectively. These values could be increased up to 52.34% ± 5.72% for DON during 2018 to 2019, to 29.64% ± 3.42% for ZEN during 2024 to 2025, or to 26.26% ± 7.89% for FB during the 2021 to 2022 harvest seasons, based on mean concentrations reported in Figure 1. Therefore, the *Fusarium*-produced mycotoxin contaminations should contribute to final diet contamination, when they are mixed with other contaminated corn forages (i.e., corn silage, high-moisture ear corn, or high-moisture corn) and concentrates. Our results also support the integration of forage-specific data into predictive models and early-warning systems by capturing occurrence patterns, environmental drivers, and the frequent co-occurrence of multiple toxins (Gallo et al., 2024). Data derived from large-scale screening further provides the basis for developing predictive equations that improve risk assessment analysis in dairy farm systems and ultimately complements chemical characterization during the screening phase, increasing both efficiency and decision-making accuracy (Battilani, 2016). Integrating these aspects into recently proposed animal nutrition models (e.g., NorFor, 2011; INRA, 2018; NASEM, 2021; and CNCPS v.6.5, as described in Van Amburgh et al., 2015) is crucial because it allows the representation of both nutritional quality and contamination risk. Assessing mycotoxin risk should not focus solely on corn silage; cereal straws and alternative silages also deserve attention, especially in years favorable to *Fusarium*. Broadening surveillance to the full forage spectrum enables a more accurate exposure assessment and better protects both animal health and milk quality.
